# Supplementary material for: Antioxidant Activity, Stability in Aqueous Medium and Molecular Docking/Dynamics Study of 6-Amino- and N-Methyl-6-amino-L-ascorbic Acid
Source: Int J Mol Sci. 2023 Jan 11;24(2):1410. doi: 10.3390/ijms24021410 (PMC9862502; doi:10.3390/ijms24021410)
Supplement: Supplementary file 1 [file ijms-24-01410-s001.zip › ijms-2092037-supplementary.pdf]

Supplementary materials

Antioxidant Activity, Stability in Aqueous Medium and Molecular Docking/Dynamics Study of 6-Amino- and *N*-Methyl-6-amino-L-ascorbic Acid

Lara Saftić Martinović [1], Nada Birkic [1], Vedran Miletić [2], Roberto Antolović [3], Danijela Štanfel [4] and Karlo Wittine [5,*]

1 Department of Biotechnology, University of Rijeka, Radmile Matejˇci´c 2, 51000 Rijeka, Croatia

2 Group for Applications and Services on Exascale Research Infrastructure, Faculty of Informatics and Digital Technologies, University of Rijeka, Radmile Matejˇci´c 2, 51000 Rijeka, Croatia

3 Department of Nursing, School of Medicine, Catholic University of Croatia, Ilica 242, 10000 Zagreb, Croatia

4 Jadran-Galenski Laboratorij d.d., Svilno 20, 51000 Rijeka, Croatia

5 Selvita Ltd., Prilaz Baruna Filipovi´ca 29, 10000 Zagreb, Croatia

* Correspondence: karlo.wittine@selvita.com; Tel.: +385-95-396-8668

(A)

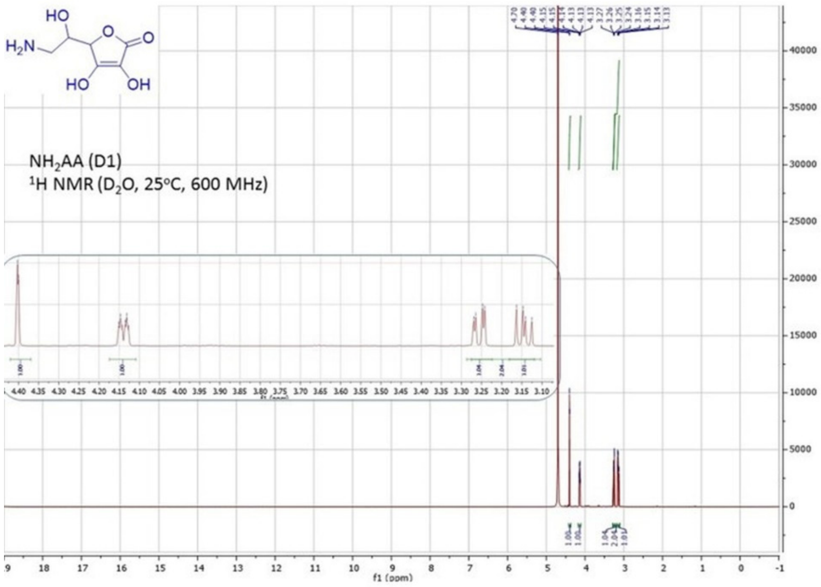

(B)

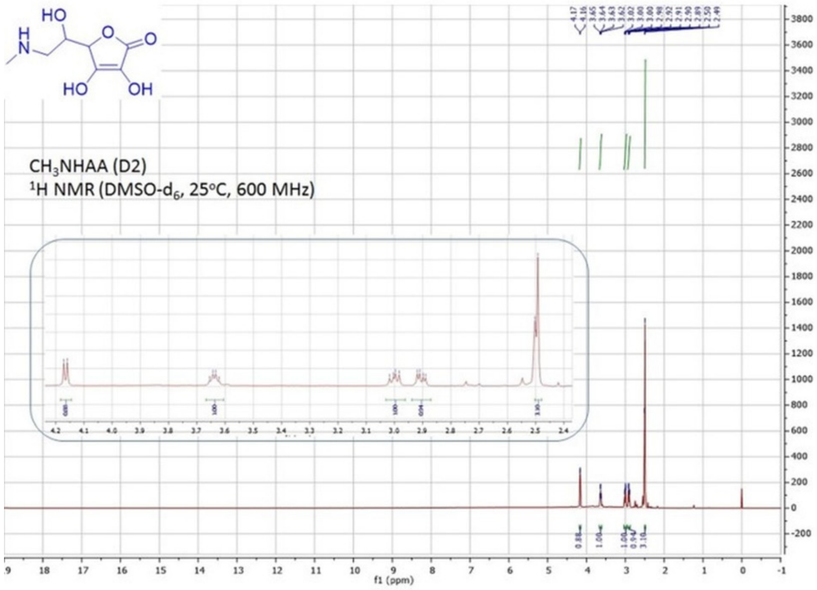

Figure S1. NMR spectrum of NH_2AA (D1) (**A**) and CH_3NHAA (D2) (**B**).

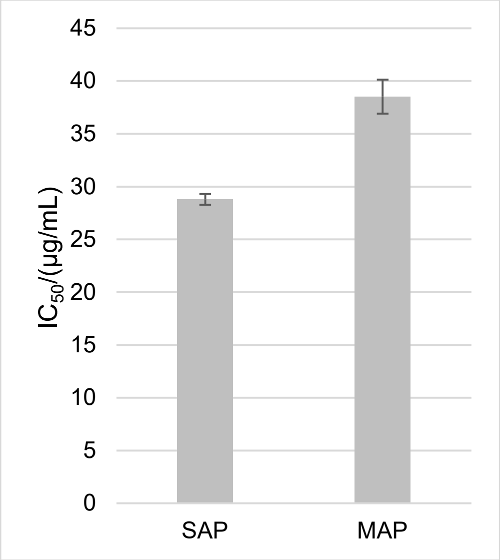

Figure S2. ABTS antioxidant activity of sodium ascorbyl phosphate (SAP) and magnesium ascorbyl phosphate (MAP) at day 0. Results are presented in µg/mL ± standard deviation.

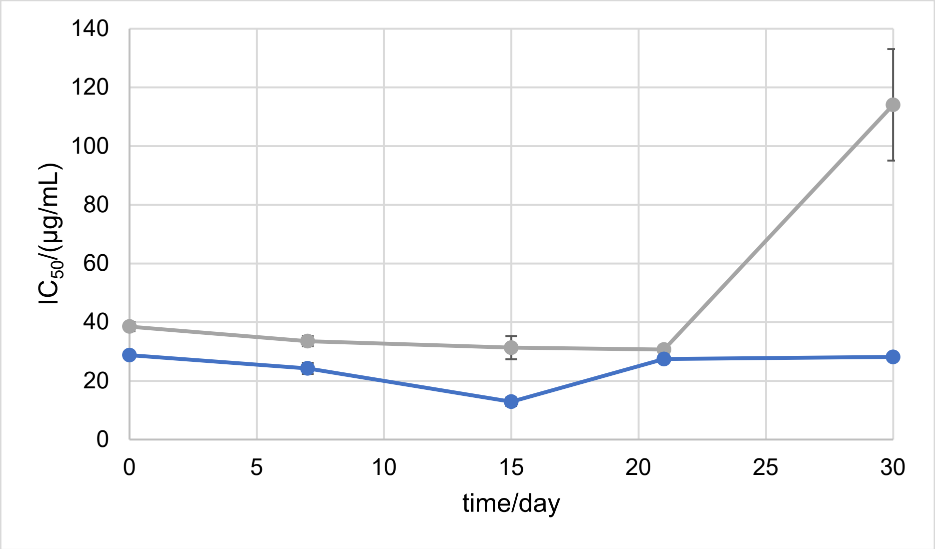

Figure S3. ABTS antioxidant stability of sodium ascorbyl phosphate (SAP) and magnesium ascorbyl phosphate (MAP) analysed after 0,7,15,21 and 30 days of incubation at 20 °C in the dark. Results are presented in µg/mL ± standard deviation. SAP derivative is shown in blue and MAP derivative in gray.

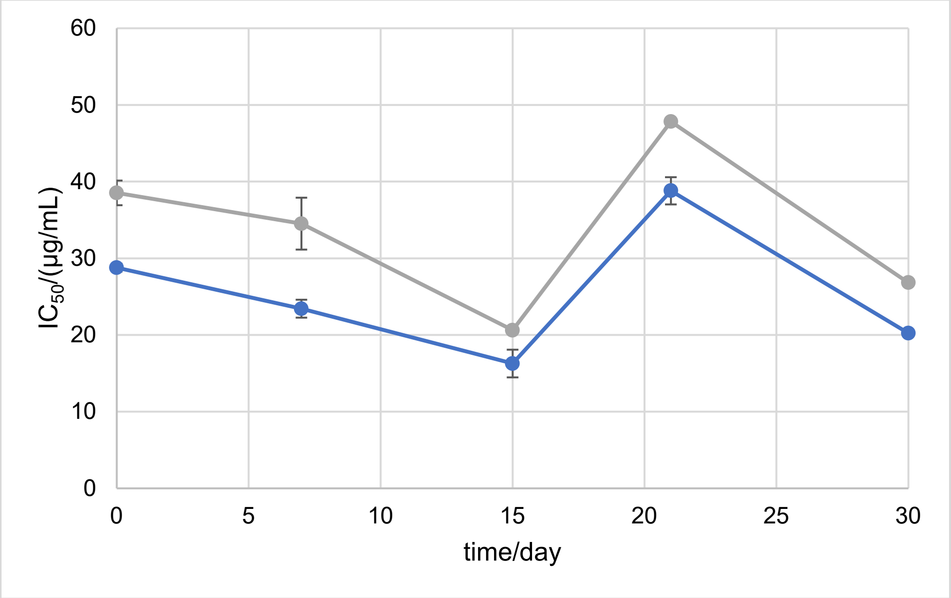

Figure S4. ABTS antioxidant stability of sodium ascorbyl phosphate (SAP) and magnesium ascorbyl phosphate (MAP) analysed after 0,7,15,21 and 30 days of incubation at 4 °C in the dark. Results are presented in µg/mL ± standard deviation. SAP derivative is shown in blue and MAP derivative in gray.

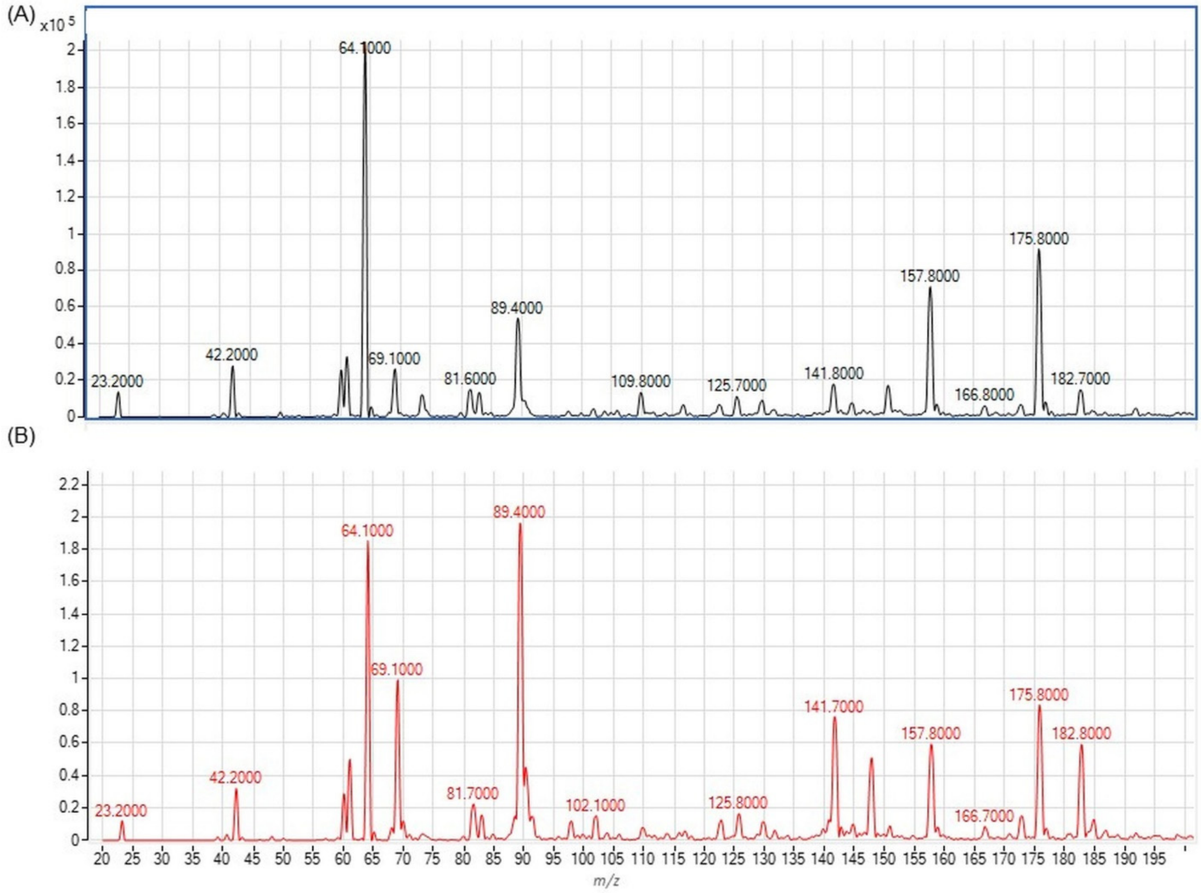

Figure S5. MS scan of NH_2AA (D1) in acidic medium analyzed on day 0 (**A**) and day 30 (**B**).

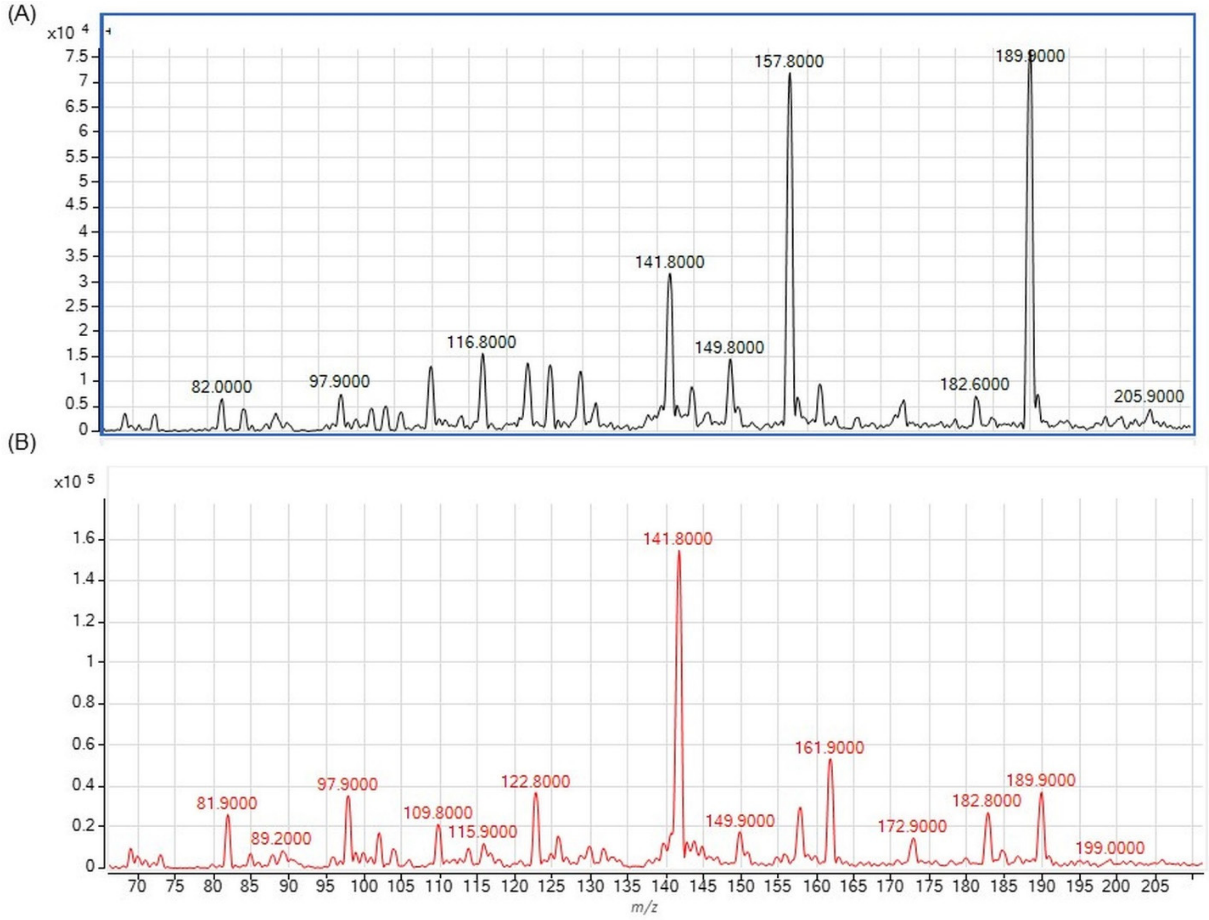

Figure S6. MS scan of CH_3NHAA (D2) in acidic medium analyzed on day 0 (**A**) and day 30 (**B**).

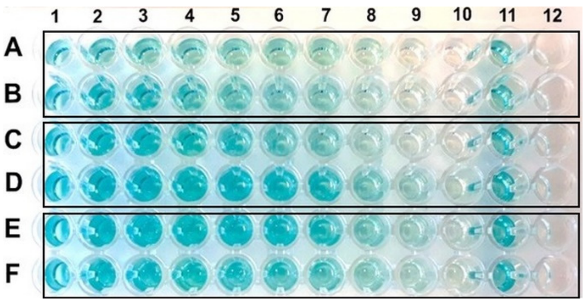

Figure S7. ABTS analysis of newly synthesized derivatives: NH_2AA (D1) and CH_3NHAA (D2) on a microtiter plate. Rows A, B, C denote derivative D1, rows D,E,F denote derivative D2. Column 11 contains a blank sample (Milli-Q water and ABTS solution for analysis), column 12 contains a blank (Milli-Q water).

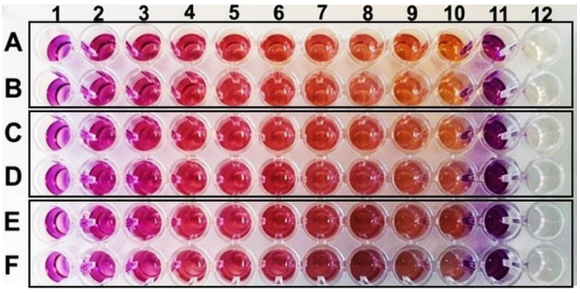

Figure S8. DPPH analysis of newly synthesized derivatives: NH_2AA (D1) and CH_3NHAA (D2) on a microtiter plate. Rows A, B, C denote derivative D1, rows D,E,F denote derivative D2. Column 11 contains a blank sample (Milli-Q water and DPPH solution for analysis), column 12 contains a blank (Milli-Q water).
